# Evolution of Robotic-Assisted Hepatobiliary Surgery

**DOI:** 10.3390/bioengineering12111221

**Published:** 2025-11-07

**Authors:** Dinh N. Nguyen, Abid Qureshi, Christina Cuoccio, Elliot G. Moore, Romulo Genato, Luca Milone

**Affiliations:** Department of Surgery, The Brooklyn Hospital Center, Academic Affiliate of the Icahn School of Medicine at Mount Sinai, New York, NY 11201, USA; dnguye12@tbh.org (D.N.N.); aqureshi@tbh.org (A.Q.); christina.cuoccio@gmail.com (C.C.); elliotgmoore90@gmail.com (E.G.M.); rgenato@tbh.org (R.G.)

**Keywords:** robotic-assisted liver surgery, evolution of liver surgery, evolution of hepatobiliary surgery, history of robotic liver surgery

## Abstract

Robotic surgery has grown tremendously in the past decade and has been applied in many surgical specialties, including hepatobiliary surgery. The complexity associated with hepatobiliary surgery initially limited the growth of implementing the use of robotics; however, technological breakthroughs made to the robotic surgical system and the superior patient outcomes have contributed to its exponential growth in the field. This article explores the evolution of robotic-assisted hepatobiliary surgery, from the advancement of the robotic surgical system to the progression of techniques applied in hepatobiliary surgery, and how this positively affected patient outcomes.

## 1. Introduction

There have been many advances in hepatobiliary surgery since the early 2000s, predominantly linked to the improvement of robotic technology. Hepatobiliary surgery is a highly advanced sub-specialty that requires vigorous training and experience in order to be performed safely. The complexity of the vascular and biliary anatomy is the main contributing factor that leads these surgeries to be laborious. Before the introduction of robotic technology, laparoscopic liver resection was considered safe and effective when being compared to open liver surgery. However, laparoscopy has many limitations that interfere with achieving the operative complexity and dexterity required for these intricate procedures. Liver surgery has evolved dramatically from the first segmental anatomy described by Couinaud in 1957, from being performed via laparoscopy in 1992 to the first robotic-assisted liver resection in 2003. The rise of robotic technology has revolutionized the field of minimally invasive surgery and alleviated the many shortcomings of laparoscopic surgery. During the International Consensus Conference on Laparoscopic Liver Resection in 2014 in Japan, the benefits of laparoscopic liver resection were discussed, such as magnified views, the exposure of sensitive structures along the hilum, caudal to cephalad views, and the effects of pneumoperitoneum on minor hemorrhages. However, these benefits are overshadowed by an inadequate range of motion due to the stiffness of laparoscopic tools and limited access to the entire liver surface, especially the posterior segments [1]. The development of robotic technology overcame the inconveniences associated with laparoscopy. The mechanical advantages of the current robotic systems are instruments, such as endowrist, that provide 7 degrees of freedom, increasing dexterity and precision of movement, with motion scaling and tremor filtering, high-resolution three-dimensional vision, and magnification of the operative field, as well as improved ergonomics. These characteristics allow for precise dissection of the tissues, intracorporeal- and micro-suturing, and easier bleeding control [2]. Therefore, robotic-assisted surgery has gained popularity over recent years and has expanded its use to include almost every area of surgery [3]. The robotic-assisted minimally invasive approach is becoming famous worldwide and potentially becoming the new standard of care for hepatic resections, biopsies, and even major hepatic surgery [4]. Comparable oncological outcomes can be maintained, as these are safely performed with less blood loss, speedier recovery from surgery, and less postoperative pain [5]. In addition, the increasing laparoscopic experience and the availability of new technologies have made this more feasible.

The adoption of robotic use for major hepatectomies was initially slower than for the use of minor hepatic surgery, but it has accelerated in recent years. Wedge resections, hemi-hepatectomies, and extended hemi-hepatectomies were among the first cases of robotic hepatectomies [6]. Due to the possibility of uncontrolled hemorrhage and the need for specialized training, minimally invasive surgical approaches for major hepatectomies have been discouraged, owing to the intricacy of the dissection of major vessels [7]. Although introducing robotic surgical equipment in hepatic surgery has changed contemporary practice, only 5.3% of minimally invasive hepatectomies in the United States were performed in 2014 [6]. The University of Pittsburgh Medical Center conducted retrospective research that looked at 1236 procedures between 2009 and 2014, where 157 of these procedures were performed robotically. Despite the low conversion rate (3.1%) and 90-day mortality rate (1.1%), the total complication rate (18.6%) and perioperative mortality rate (91%) were high. Between 2007 and 2010, Buchs et al. [6] conducted a similar study at the University of Illinois Hospital and concluded that robotic hepatic surgery could be performed safely with low morbidity and mortality. However, several risk factors have been identified as independent causes of higher morbidity and mortality; this highlights the crucial role in patient selection [8]. Due to hepatic surgery being regarded as a more complex procedure, it correlates with a higher risk of complications, morbidities, and mortalities [9]. Although early reports on robotic liver surgery focused primarily on minor resections [10], multiple cases of robotic major hepatectomies have been published to date. Several factors were independently associated with increased postoperative morbidity, encompassing both patient- and procedure-related variables. Patient-related risk factors included cardiovascular and renal comorbidities, an American Society of Anesthesiologists (ASA) score ≥ 3, age ≥ 70 years, and the presence of malignant disease. Procedure-related risk factors included estimated blood loss ≥ 500 mL, intraoperative transfusion, multiquadrant surgical involvement, and advanced procedural complexity [6]. Multivariable analysis identified advanced procedures, multiquadrant operations, malignant disease, body mass index (BMI) < 30, hypertension, and transfusion as being significantly associated with an increased risk of postoperative complications [6]. Furthermore, an ASA score ≥ 3, age ≥ 70 years, cardiovascular comorbidity, and blood loss ≥ 500 mL were significantly associated with increased postoperative mortality [6].

In 2023, the World Journal of Gastroenterology [11], via an international panel of experts, published guidelines on indications for robotic liver resection. Among those, the indications were similar to laparoscopic and open surgery, such as hepatocellular carcinoma, cholangiocarcinoma, colorectal liver metastasis, benign hepatic tumors, and living donor hepatectomy. For the above mentioned indications, the robotic approach was deemed safe in comparison to laparoscopy or open approaches. For tumors in close proximity to major vessels or biliary structures, the robotic approach was indicated if parenchymal free dissection could be obtained or these structures would be needing excision and reconstruction. Robotic-assisted liver surgery may also be helpful in staged liver partition with single or multiple procedures, as an option for patients with insufficient residual liver volume, allowing for locoregional and systemic therapies [12].

**Figure 1:**
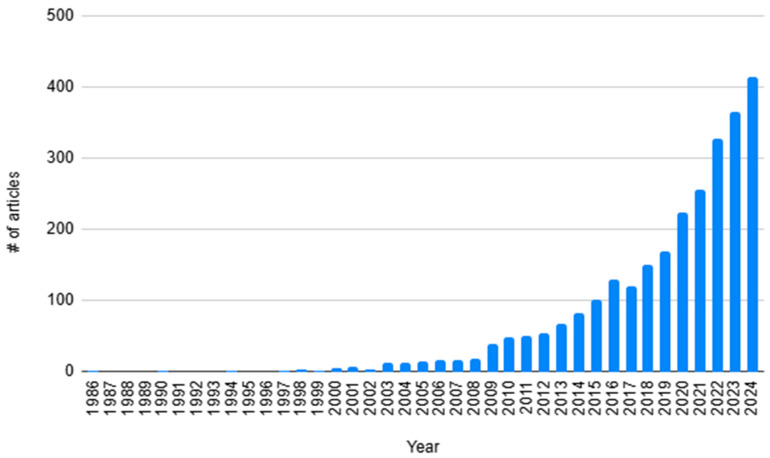
A search on PubMed for ‘Robotic Liver Surgery’ showed a rise in publications in robotic-assisted hepatobiliary surgery from 1986 to 2024.

**Figure 2:**
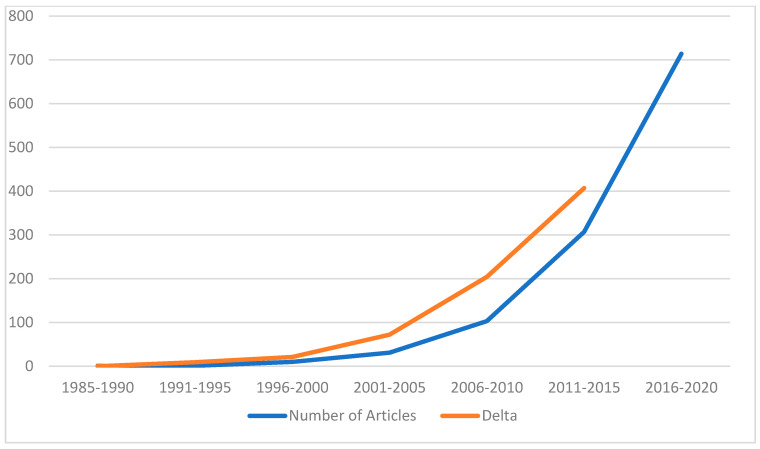
Number of articles and the rise in publications.

Through literary searches for robotic liver surgery alone, the steady rise in publications can be seen in robotic-assisted hepatobiliary surgery over the last four decades (Figure 1 and Figure 2). This paper aims to explore the history of robotic-assisted hepatobiliary surgery and discuss how the changes in the technology of the robotic systems have affected surgeons’ techniques and patient outcomes. We will also discuss the future of robotic surgery and how the development of artificial intelligence may benefit the field of minimally invasive surgery.

## 2. Materials and Methods

### 2.1. Da Vinci System

The Da Vinci robot-assisted surgical system is the most advanced robot-assisted surgical system used globally. It was developed by the American company Intuitive Surgical and was launched on the market after FDA approval in 2000. It has been applied in various surgical fields, including hepatobiliary surgery. The Da Vinci robotic system is the first FDA-approved system that includes surgical instruments all with cameras and scopes, in comparison to previous surgical robots that depended heavily on endoscopes and numerous surgical assistants to perform surgery [13]. Da Vinci comprises three subsystems: (1) a surgeon console, (2) a patient cart, and (3) a 3D vision chart. The 3D magnification screen allows the surgeon to view the operative area with high-resolution clarity.

In 2019–2020, there were 4986 Da Vinci surgical system units worldwide—2770 in the United States, 719 in Europe, 561 in Asia, and 221 in the rest of the world. More centers have adopted robotic-assisted surgery, hoping to improve the limitations of laparoscopy, as robotic-assisted surgery offers technical benefits such as seven degrees of freedom and 3D visualization. Despite the advantages of the Da Vinci robot, there are still controversies that reduce its use worldwide. The costs of the Da Vinci significantly reduce its availability and use [14]. In addition, the machine’s size poses concern as it requires larger surgical rooms to fit the robot and other surgical equipment with enough additional room for the surgical team. Additionally, surgeons require specialized training and experience to fully utilize a system that requires a unique and specific skill set.

### 2.2. The Evolution of the Robotic System

**Figure 3:**
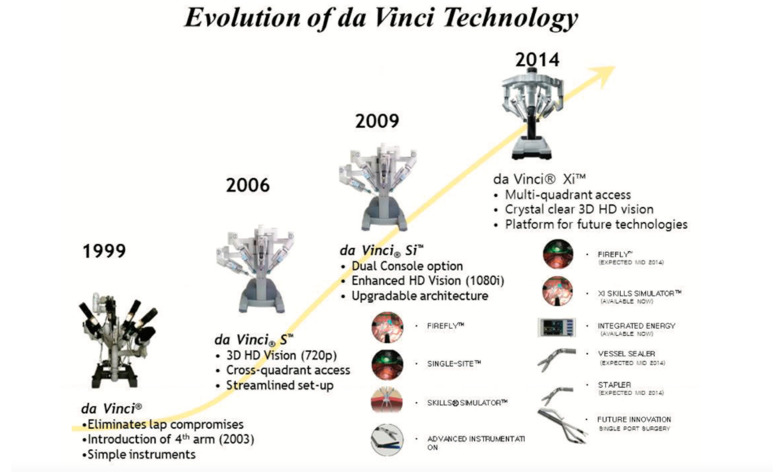
Evolution of the Da Vinci surgical system (Intuitive Surgical, Sunnyvale, CA, USA).

The first-generation Da Vinci surgical system (Intuitive Surgical, Sunnyvale, CA, USA) was approved in 2001 and consisted of three robotic arms. The second-generation Da Vinci “S” was released in 2006, and this model introduced a fourth robotic arm. The Da Vinci Si H.D. (3rd generation) introduced a dual console and many other features such as Firefly to enhance visualization, a simulation option to assist in education, and new instrumentation. The robotic arms on the third-generation Da Vinci came out of the midline of the robot. In 2014, the FDA approved the fourth-generation Da Vinci, “Xi.” It features a redesigned surgical arm cart and smaller and longer arms, with the arms coming out of the system’s top. The Xi model also added a new camera system, allowing flexibility for cart position and port placement. In addition, the robotic arms are smaller and more slender, approximately 1 cm in diameter, with redesigned joints that allow for a greater range of motion. The smaller operating arms in the Xi model avoid leveraging the sides of the incision walls, removing contact between the robot and the interior tissue. This advantage reduces the risk of infection. The endowrist addition precisely replicates the surgeon’s movements at the controls, improving accuracy in small operating spaces. The evolution of Da Vinci technology is shown below in Figure 3.

There are many differences between the Si and Xi models. The most evident is the design, where the robotic arms come out of the midline in the Si model, whereas the robotic arms come out of the top in the Xi model. The Si model can only support a camera system in robotic arms labeled 2 and 3, whereas the Xi model can support the camera system in any robotic arm [15]. The addition of the Firefly imaging feature was added to the Si model. This innovation was designed to assist in the visualization of the hepatobiliary structures [15]. Firefly is a laser light source used to excite the fluorophores at wavelengths around 800 nm, and the emitted light is captured by the image sensors on the endoscope [16]. Fluorescent imaging uses intravenously administered Indocyanine Green (ICG), and when stimulated by polarized light, it allows for visualization of the vascular and biliary structures. ICG is a water-soluble, tricarbocyanine dye with peak spectral absorption at 800 nm and is mainly bound to albumin (95%), allowing the dye to remain in the intravascular space. It has a short half-life of 2 to 5 min, is metabolized by the hepatic parenchymal cells, and is secreted entirely into the bile. This allows surgeons to have high-resolution vision of near-infrared images of blood flow, organ perfusion, and biliary structures. It visualizes structures by detecting indocyanine green (ICG) dye fluorescence, helping to differentiate tissues in real time. Firefly enhances the identification of critical structures such as bile ducts, lymph nodes, and blood vessels. This allows surgeons to make more precise dissections and avoid injury to vital anatomy. In hepatic surgery, Firefly assists in segmental mapping, guiding precise parenchymal transection along functional boundaries. In oncologic procedures, Firefly can delineate tumor margins or sentinel nodes, influencing decisions about the extent of resection. It can also reveal segmental boundaries and assess remnant liver perfusion, affecting the surgeon’s choice of resection plane.

The Xi system significantly improves joint setup accuracy compared to the Si [17]. A study by Ferguson et al. [17] revealed that the Xi system would not require additional tracking protocols such as optical tracking. The Xi is remarkably accurate and achieves sub-millimetric errors after calibration, with the mean error being 2 mm. Calibration of the robot can improve accuracy, but the Xi model has an excellent baseline accuracy. Lack of feedback capabilities on the Da Vinci surgical system require surgeons to rely solely on visual cues for estimating interaction forces with tissue. This reliance on visual force estimation presents a significant learning challenge and typically demands extensive experience to master. Notably, expert surgeons often report a subjective ability to ‘feel’ tissue interaction forces through visual interpretation alone [18].

The endoscopic linear stapler, endowrist Staplers 30 mm and 45 mm, received its clearance for the Da Vinci Si and Xi in the United States in October 2012 and July 2014, respectively [9]. This new technology offers full wrist articulation in both horizontal and vertical planes. It mimics human wrist motion and improves access to difficult-to-reach anatomical areas. The wristed articulation allows for the precise placement of staplers in narrow anatomical corridors, which can influence decisions regarding resection technique and angle. Real-time feedback on tissue compression ensures safe and effective stapling, reducing intraoperative hesitancy or the need for repeated adjustments.

The surgeon receives feedback throughout usage via SmartClamp technology, which lessens the element of guessing usually involved in laparoscopic stapling [9]. SmartClamp technology can determine if the jaws can sufficiently close on the target tissue for the specified staple height for each reload color and alerts the user if they cannot [9].

The SureForm brand of staplers became available on the market in 2018. Its fundamental component is SmartFire technology, which automatically adjusts to optimize the staple line while monitoring tissue compression before and during firing. The ability to approach tissue where it naturally lies is made possible by the 120-degree cone of articulation, potentially eliminating the need to pull tissue toward the stapler. Data from March 2019 reveals that the SureForm robotic stapler employs a wider range of motion than the human hand or the industry’s top laparoscopic staplers, including the Endo GIA Ultra with Tri-Staple technology, SIGNA with Tri-Staple 2.0 technology, and Powered Echelon with GST [19]. According to statistics from Intuitive Surgical, as of March 2019, SureForm staples had a 98.7% higher rate of being optimally formed across a spectrum of tissue thickness > 4 mm [19]. Both the 45 mm and 60 mm devices were tested using black reloads on in vivo porcine stomach tissue. SureForm 60 mm performs noticeably better in thick tissues than Endo GIA and SIGNIA [19]. In thick tissues (>4 mm), the SureForm 45 mm performs noticeably better than all three staplers. SureForm also has a 100% success rate in tissue approximation with less outer layer tearing, which is significantly better than that of Echelon Flex [19]. The SmartFire technology’s automatic measurement and adjustments during firing deliver a more consistent cut line length across thin and thick tissues, allowing for greater tissue control and preserving the cut line’s integrity. This technology also aids in achieving the expected length of transection, avoiding unexpected reloads and simultaneously lower costs. Compared to Echelon Flex, Endo GIA, and SIGNIA, surgeons utilizing SureForm staplers applied much less target tissue tension during positioning and clamping [19].

### 2.3. Evolution of Liver Dissection Technique

The most reported application of the Da Vinci robot in the early years was for so-called minor or simpler procedures, including minor liver resection, wedge resection, and anterolateral/peripheral segmentectomy (37.7%) [20].

With the improvement on the machine reported above, the robotic-assisted hepatobiliary surgery has expanded to include liver transplant and oncologic treatments. In the beginning, the main challenge was the transition from a laparoscopic to robotic approach, since most of the first uses were still in minimally invasive surgeries. The techniques for port placement in robotic-assisted surgery are slightly different from the laparoscopic technique [21]. Five or six ports are used in robotic-assisted surgery, consisting of four robotic arms, including one for the camera and one to two for the bedside assistant surgeon [16].

The robotic procedure for right hepatectomy is probably the most standardized technique and the most important to report for the evolution of robotic hepatobiliary surgery. It is mainly divided into three main steps: hepatocaval dissection, dissection of the hepatic hilum, and parenchymal transection [1]. Parenchymal dissection is the most challenging part of robotic liver resection. There have been advancements in robotic technology that improve surgical skills, which include intuitive hand-control motions, tools with seven degrees of freedom, a magnified three-dimensional perspective, and consistent robotic arm retraction. There are currently no standards for parenchymal transection, but many techniques have been described. The current robotic instruments for parenchymal transection have several limitations, and there is no standardized technique for this step. Through advances in the robotic system and the addition of new tools, some techniques have been modified to render this historically complex process less arduous. Hence, we report here the evolution of parenchymal dissection over the years with the pros and cons of the techniques.

### 2.4. Techniques for Parenchymal Dissection

#### 2.4.1. Initial Traction Method

The initial dissection of the liver parenchyma is along the cholecysto-caval line on the anterior surface of the liver [22]. This part of the procedure is accomplished using a combination of robotic harmonic shears (Intuitive Surgical, Inc) on the right arm and bipolar forceps on the left arm. Multiple deeper layers of polypropylene stay sutures are used to achieve optimal exposure during liver transection. Hemostasis is maintained using bipolar cautery for small and minor bleeds, while polypropylene 4-0 sutures are utilized for larger bleeds.

The second phase of parenchymal dissection is the most complex part of the surgery. It involves sectioning the core of the liver parenchyma, which has rich vasculature from venous branches of segments V and VIII directed towards the middle hepatic vein. Laparoscopic endoclips can also be used for this step. Before the invention of the robotic stapler, the transection of the right hepatic vein from the parenchyma was performed via the assistant port with a laparoscopic vascular stapler. The lateral ligaments of the liver are typically taken entirely down at the end of the procedure. The specimen is extracted via a Pfannenstiel mini-laparotomy. Two closed suction drains are left in the subhepatic and subdiaphragmatic areas.

The Initial Traction Technique, which was created and evolved by Giulianotti [22], shows that the procedure is safe and effective. The conversion to open rate is 4.2% (1 patient). The mean operative time was 337 min, ranging from 240 to 480 min. The mean intraoperative blood loss was approximately 457 mL and ranged from 100 to 2000 mL. Three patients (12.5%) required blood transfusion postoperatively. There was no mortality noted postoperatively, and none of the patients required reoperations. However, six patients (25%) experienced postoperative morbidity, which included transitory liver failure in two patients and pleural effusion, bile leak, fluid collection, and deep venous thrombosis in one patient. Giulianotti et al.’s [23] findings show that zero mortality and low morbidity indicate that robotic right hepatectomy is safe and feasible when performed by experienced surgeons. Their results at the 34-month follow-up confirm the procedure’s effectiveness due to no evidence of port-site metastases in patients with malignant pathologic findings.

With the advances in the Da Vinci robot surgical system and the development of new tools, Giulianotti modified his technique to overcome some of the limitations of laparoscopy while using the robot. Transection of the parenchyma is performed layer by layer using the harmonic shears or the Habib from the cortical aspect of the liver towards the core of the parenchyma. Stay sutures should be placed on the left side of the transection line and held by the fourth robotic arm to exercise traction and better exposure of the hepatic surface. The residual parenchyma and the left hepatic vein are divided using a robotic vascular stapler. The surface of the remnant liver is controlled, and the specimen is extracted. Two closed suction drains are placed around the resected area [23].

#### 2.4.2. Rubber Band Suspension Method

The rubber band suspension method uses the harmonic scalpel on one robotic hand and the Maryland forceps or the endowrist vessel sealer on the other. The initial traction method was used as the technique by Giulianotti et al. [20,21]. The placement of the harmonic scalpel usually depends on the direction of parenchymal transection, since this robotic instrument lacks endowrist capabilities. In sectionectomy or hemi-hepatectomy, the parenchymal transection plane is formed vertically. Because of the curved shape of the tip of the harmonic scalpel, holding the harmonic scalpel in the left hand can make the tip’s direction identical to the parenchymal transection line.

The rubber bands are usually sutured in place, on each side of the transection line, on the surface, and in progressively deeper levels of the liver parenchyma, and then secured to the abdominal wall with a mosquito clamp. This allows the camera to be kept in a central view while opening the parenchymal resection plane. Using rubber bands helps to avoid injury to the hepatic tissues, opens the transection plane gradually, and obviates the need for an additional surgical assistant.

A study from Yonsei University Health System in Seoul, Korea, consisting of 69 patients, from December 2008 to May 2016, using the rubber band method with the Da Vinci robotic system, showed that the median operation times of major hepatectomy was 518 min, and that of minor hepatectomy was 360 min [24,25]. The median estimated blood loss was 200 and 100 mL in major and minor hepatectomy, respectively. Six patients required perioperative transfusion and six required conversions to open surgery. The reasons for conversion were bleeding; technical difficulty in the dissection of the liver hilum; tumor adhesions to the diaphragm; poor exposure and long operation time; and injury to the left bile duct during living donor right hepatectomy. Open conversion was usually performed in the late stage, and the operation was completed only through a midline mini-laparotomy. The postoperative complication was assessed according to the Clavien–Dindo classification. The overall complication rate was 43.5%, and grade III complications only occurred in seven patients. The median length of stay in the hospital was 8 days and ranged from 5 to 46 days. Oncologic outcomes between the minimally invasive and open surgery groups showed similar outcomes regarding resection margin and disease-free survival. The 5-year disease-free survival was 40.2% in the minimally invasive group and 50.5% in the open group. Among 99 patients who underwent minimally invasive liver resection, 16 received robotic liver resection. Major hepatectomy and anatomic liver resection were more frequently performed in the robotic group than in the laparoscopic group. There were no oncologic outcome differences when comparing patients who received laparoscopic left sectionectomy liver resection to patients who underwent robotic resection.

This rubber band suspension method has several advantages. The elastic power of rubber bands can automatically expose the parenchymal transection plane [26]. Also, all three robotic instruments can be used during parenchymal transection. The primary working arms are the first and second; therefore, the third robotic arm can work as an assistant in open surgery. The third arm can be used to compress active bleeding or further expose the deep or inferior portion of the liver parenchyma. This technique enables the operator to be less dependent on the assistant during parenchymal transection.

This technique has several disadvantages; therefore, it cannot be defined as the gold standard for robotic liver surgery. The main limitation of harmonic scalpel transection is the lack of endowrist function, increasing the risk of serious injury to intrahepatic vessels [27]. Due to the lack of endowrist function, another limitation is that the harmonic scalpel is challenging to use to track various parenchymal transection planes. Therefore, other techniques and tools must be developed to foster robotic liver surgery. The harmonic scalpel still poses potential risk for partial injury of large vessels in the deep liver parenchyma. It has a limited effect on controlling bleeding from the moderate-sized portal pedicle and healing the bile duct [25,26]. A new endowrist vessel sealer has been introduced and is thought to be useful for parenchymal transection; however, due to the bulky head, it seems to have limitations in effective transection of liver parenchyma. There are other limitations for severe adhesions to or tumor invasion of the diaphragm which make it challenging to dissect the right liver from the diaphragm due to the rigid form of the telescope and the shaft of the instrument [26]. Flexible telescopes and more flexible instruments should be developed to overcome these limitations.

#### 2.4.3. Three Devices (3D) Technique

A study performed by Perrakis et al. [27] studied a technique, the ‘3D (Devices) technique,’ for parenchymal transection. Their study included 28 patients using the Da Vinci Xi, consisting of 3 main instruments: monopolar scissors, the robot’s bipolar Maryland forceps, and a laparoscopic-guided waterjet. Of the 28 cases studied, 12 were major hepatectomies, and 16 were minor hepatectomies for malignant and non-malignant causes.

Robotic arm 2 of the Xi System was utilized to hold the camera, which was positioned directly above the umbilicus. For the Pringle maneuver, a 10 mm trocar was inserted into the patient’s right side, and a second 10 mm trocar was added for laparoscopic support on the left, or supraumbilical, side.

Controlling the hepatic inflow is the initial step in a significant liver resection. An energy device, such as a tiny vessel sealer, is used to achieve hemostasis in small vessels less than 7 mm in diameter. Alternatively, vascular staplers, clips, or ligatures are used to control bigger vessels (>7 mm in diameter) [27]. The left or right bile duct can be cut extra- or intra-hepatically. The left hepatic vein can be exposed, clipped, and cut for a left hemi-hepatectomy. Due to the increase in difficulty when accessing the right hepatic vein when compared to the left, it is often transected toward the completion of the parenchyma phase. In significant, and occasionally, even minor liver resections, the liver is removed from the inferior vena cava once inflow and outflow are regulated.

Parenchymal dissection is the next stage after vascular control is accomplished. At the surface of the liver, the resection margin is marked with monopolar scissors under ultrasound guidance to ensure the correct plane. During the transection, ultrasound can be utilized to verify the plane [2]. The monopolar scissors (robotic arm 1) and the bipolar Maryland forceps are operated by the console surgeon (robotic arm 3). The 10 mm periumbilical port is used by the assistant to control the waterjet on the patient table. Parenchymal dissection is made possible due to the simultaneous employment of the three devices, thus the name “3D method”. The waterjet aids in the identification of intrahepatic arteries and bile ducts with extreme accuracy, care, and control, which then allows for safe ligation of these structures. Small vessels are coagulated using the bipolar forceps. The fundamental benefit of the waterjet is that the saline aggravates the coagulation process of the bipolar forceps, further decreasing blood loss. Additionally, the constant flow of saline keeps the instruments free of coagulated and necrotic tissue, and the extra fluid be suctioned out to make visualization easier. The monopolar scissors remove hepatic tissue from the intrahepatic structures and support the efforts of the waterjet.

Their results show that the operative time for major liver resections was 381.7 (SD 80.6) min and 252.0 (SD 70.4) min for minor resections. Intraoperative measured blood loss was 495.8 (SD 508.8) ml for major and 256.3 (SD 170.2) ml for minor liver resections. For all cases, the mean postoperative stay was 13.3 (SD 11.1) days. Hepatic surgery-related morbidity was 10.7%, and no mortalities occurred. There was an R0 resection in all malignant cases. Perrakis et al. [27] concluded that their novel 3D technique for parenchymal dissection in robotic liver surgery is safe and feasible. Their novel method offers an advanced locally controlled preparation of intrahepatic vessels and bile ducts.

This method requires the bedside assistant to be skilled and experienced in handling the waterjet. Since the waterjet must come into intimate contact with the liver tissue during parenchymal dissection, the console surgeon and the bedside assistant must collaborate closely and uniformly. Water reflection on the camera is unfavorable when the waterjet is distant from the liver parenchyma, which negatively affects the visual field and necessitates frequent cleaning of the camera. Another drawback of the method is that in cases where fibrosis or cirrhosis is present, parenchymal dissection using the 3D method takes longer when compared to other parenchymal dissection techniques using other instruments. However, Perrakis’ findings demonstrated that their method enabled precise liver parenchyma dissection while minimizing blood loss. Another drawback of this technique is the lack of endowrist qualities of the waterjet, which restricts the instrument’s flexibility and rotation. However, when compared to other techniques, Perrakis’ 3D technique enables superior local control and a better exposition of the intrahepatic arteries and bile ducts.

#### 2.4.4. Saline-Linked Monopolar Cautery Scissors (SLiC-Scissors) Technique

A study performed by Fujikawa et al. [28] developed and studied a novel technique for parenchymal transection called saline-linked monopolar cautery scissors (SLiC-Scissors). Their study used the Da Vinci Xi, and included ten cases, four being of colorectal cancer liver metastasis and six of malignant liver tumors. Three patients had liver cirrhosis, and two had liver metastases from colon cancer that required hepatectomy following chemotherapy and dissection of the primary lesion. This technique requires five trocars, four of which are the robotic arms, and the fifth trocar is for the assistant surgeon. Two surgical assistants are needed for this technique, with one assistant standing on the patient’s left side and the other standing between the patient’s legs.

For the dissection of right lobe tumors, the patient is positioned supine with the legs apart in a 10-degree reverse-Trendelenburg position, or in a left semi-flank position, to remove right posterosuperior segment tumors. The Hasson technique was used to establish pneumoperitoneum after inserting the first trocar in the supraumbilical region. Robotic arm 3 is utilized for liver retraction, robotic arm 1 is used for the Cadiere or Tip-up Forceps, robotic arm 2 is for the fenestrated bipolar forceps, and robotic arm 4 is for the Maryland Bipolar forceps. However, for parenchymal transection, the left hand will utilize the Endowrist Suction Irrigator, while the right hand will utilize the monopolar curved scissors.

In order to perform liver parenchymal transection, the console surgeon’s forceps employ a Suction Irrigator for the left hand (robotic arm 1), Monopolar cautery curved scissors for the right hand (robotic arm 4), and Tip-up or Cadiere Forceps to tow and fix the third arm (robotic arm 1). The sterile 0.9% saline bottle was connected to an electrosurgical VIO device, and the drip rate was set at 1–2 cc/min, which was administered by the assistant surgeon using the ball-tipped SLiC. Intraparenchymal arteries under 2 mm in diameter were cut with scissors to maintain hemostasis during parenchymal transection; however, vascular structures larger than 2 mm in diameter were cut with ultrasonic coagulating shears by the assistant surgeon. The waterjet scalpel was utilized as an additional tool to the assistant surgeons when dissecting major Glissonean pedicles to prevent thermal damage to the bile ducts. The superficial layer of the dissection surface was heat coagulated at low temperatures (up to 100 degrees Celsius or below) by dripping saline droplets, carried out by the assistant, facilitating dissection and hemostasis. The surface of the parenchyma was advanced using the endowrist monopolar cautery scissors, while the saline dripping assisted with coagulation. This technique allows for fast dissection and hemostasis through heat coagulation via saline droplets. Additionally, the saline droplets allow the instruments to remain clean, saving time that would be spent removing instruments or cleaning necrotic or coagulated tissues.

The median size of the target lesions in their analysis was 25 mm, ranging from 14 to 43 mm. Their results using this method show that the median operating time was 223 min (143–433), and the console time was 134 (72–261). The median estimated blood loss was 5 mL (5–30 mL). All 10 cases were performed robotically, and no cases required conversion to open. No severe complications or deaths were noted throughout the entire postoperative period. The average hospital was seven days (4–13). The SLiC-Scissors approach for liver parenchymal transection in RLR is thus a viable and safe procedure. This technique allowed Fujikawa et al. [28] to visualize the intrahepatic arteries better; therefore, local control is enhanced with this novel method.

After analyzing the short-term results, Fujikawa et al. [28] found that the SLiC-Scissors approach is safe and efficacious for liver parenchymal transection in RLR. The median operational time was 223 min, and the console time was 134 min. The median intraoperative estimated blood loss was 5 mL (5–30 mL). No liver resections were converted to open surgery. There were no significant postoperative complications or deaths, and the median length of the recovery period was seven (4–13) days.

The current research has certain limitations. Because this study was performed retrospectively, with a small sample size (n = 10), it is limited in determining how treatment affects the outcome. In addition, a control group is needed to demonstrate which technique outperforms the other techniques. However, this novel method is a step towards standardizing parenchymal dissection.

#### 2.4.5. Six-Port Double Bipolar Clamp-Crush Technique

Egawa et al. (2024) [29] made the effort to evaluate the double bipolar clamp-crush technique in robotic liver resection to prevent the obstacles of adhesion of crushed liver tissue and carbonization in the forceps, to maximize hemostasis. Although used in open surgery in the past, this group was the first to implement it in a robotic setting. During the parenchymal dissection, the console surgeon utilizes the double bipolar method to clamp and crush the liver tissue and eliminate vasculature. The novelty of this technique is shown through the assistant surgeon using two ports to insert a ball-type electrode with a small tube to drip saline solution, while also aspirating the crushed liver tissue and blood via an endoscopic aspiration system.

The principles of this technique are similar to the Three Devices Technique mentioned earlier. The benefit of this technique is to remove the tissue and blood to negate any material sticking to the robotic forceps, and simultaneously maintain a moist surgical field to help attain a good liver parenchymal dissection. The use of the assistant ports are also helpful in providing an additional device to ensure hemostasis in addition to the console’s robotic bipolar forceps. Having an assistant operate with multiple ports allows for 2 other devices to provide irrigation and suctioning capabilities, with saline dripping. The assistant ports also were advantageous, with minimal change in robotic instrumentation. The disadvantages are the requirement of an assistant experienced in laparoscopic surgery, with the ability to coordinate with the head surgeon. There may also be interference between the surgeon robotic ports and assistant ports.

The limitation of this study was that it constituted a small sample size, with a total of 13 patients. However, the median blood loss in all 13 cases was between 5 and 256 mL. The median operative times with this technique were 211–485 min. There were no conversions to open surgery, and the study also reported no perioperative complications, indicating the effectiveness and safety of the method. Although a novelty for robotic-assisted liver surgery, the technique does show relative success and promise [29].

#### 2.4.6. Meta-Analysis

**Figure 4:**
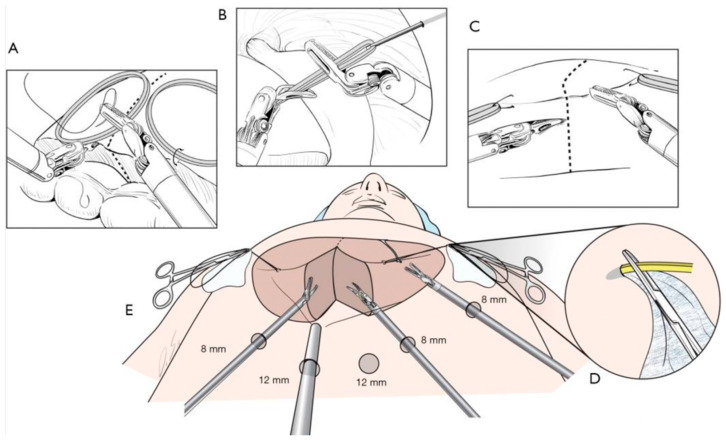
Drawing of the rubber band suspension method [25]. (**A**) Stay sutures used to fix the resection margins, (**B**) One end of the rubber band is pulled out and fixed on abdominal wall, (**C**) traction applied for transection (**D**) area covered by gauze to prevent leakage of CO_2_, where the rubber band is pulled out, (**E**) configuration of port sites.

As the popularity of robotic-assisted hepatobiliary surgery increased, physicians sought to understand the impact of this new technology on healthcare outcomes and costs. Peer-reviewed clinical publications and evidence-based medicine have become increasingly important, hence the exponential increase in publications in robotic-assisted hepatobiliary surgery in the past two decades (Figure 4). A PubMed search using the keywords ‘robotic liver surgery’ has shown an exponential rise in the number of publications on HBP surgery. Due to the increasing demand for robotic surgery, residency training has started implementing robotic-assisted surgeries as part of its curriculum. There has also been increased fellowship training in hepatobiliary surgery and transplant surgery using the Da Vinci surgical system. As of 2016, there are 26 hepatobiliary fellowship training programs in the United States and Canada.

A meta-analysis by Machairas et al. (2019) [30] compares the short-term outcomes between robotic-assisted liver surgery (RLR) and open liver surgery (ORL). It consists of 10 non-randomized retrospective clinical studies published between 2014 and 2018 and included 1248 patients (458 in RLR, 790 in OLR). Their analysis was significant for lower overall morbidity and length of hospital stay in the RLR group, which decreases overall healthcare costs. Open liver surgery was significantly associated with shorter operative time. There were no statistically significant differences in estimated blood loss (EBL), blood transfusion, R0 resection rate, or mortality rate (0.5% RLR, 0.4% OLR). The conversion to open rate was 4.6%, ranging from 0% to 8.8%. The overall morbidity rate is significantly lower in the RLR group (15.5%) compared to the open group (22.2%); however, there was no significance between the two groups in major adverse events (Clavien–Dindo III–IV) (3.2% RLR, 5.5% OLR), but there was significance for minor morbidities (Clavien–Dindo I–II), 11% RLR, and 15.9% OLR. No significant bile leaks (RLR 2.2%, OLR 2.7%) and no significant bleeding (RLR 2.2%; OLR 2.9%) were noted between the two groups. There was also significance in longer operative times in RLR, with a mean difference of 65.91 min, and length of hospital stay, with a mean difference of −2.76 days.

Ziogas et al. (2021) [31] performed a meta-analysis to compare perioperative and postoperative outcomes between laparoscopic major hepatectomy (LMH) and robotic major hepatectomy (RMH), which included seven retrospective cohort studies comparing laparoscopic and robotic interventions. Major hepatectomy is defined as resectioning three or more Couinaud segments for any underlying etiology. Of the seven studies, three in the United States, two in Italy, one in South Korea, and one in China, that were included in the meta-analysis, four were published in 2019; however, the study periods ranged from 2005 to 2018. Their meta-analysis showed that LMH and RMH have equivalent perioperative and postoperative outcomes, and their results showed no statistical significance. Note that while all these results showed differences between the two groups, it should be emphasized that these differences are not statistically significant. The overall complication rate for LMH is 28.3%, while that of the RMH group is 18.4%. The severe complication rate is defined as Clavien–Dindo grade 3 or higher, and the RMH group also revealed a lower complication rate than LMH, at 3.6%, and 6.7%, respectively. The overall mortality rate for the LMH group was 0.3%, and for the RMH group it was 0%. The conversion to open rate for LMH was 6.4% and for RMH, 3.7%. The margin positive resection rate for the LMH group was 10.1%, and for the RMH group, 6.1%. The transfusion rate for the LMH group was 14%, and for the RMH group, 18.4%. RMH and LMH are equivalent regarding all outcomes if they are performed on carefully selected patients by surgeons with surgical expertise in minimally invasive hepatic surgery in high-volume centers. Fruscione et al.’s (2019) [32] results show that patients that underwent robotic-assisted hepatectomy were less likely to be admitted to the ICU (43.9%) compared to the laparoscopic group (61.2%). Patients were also less likely to be readmitted within 90 days in the robotic group (7%) vs. the laparoscopic group (28.5%).

Similarly, Cortolillo et al. [33] conducted a nationwide analysis of outcomes and costs comparing robotic, laparoscopic, and open hepatectomy. Their analysis included 10,870 patients who underwent hepatectomy between 2013 and 2014, of which 724 patients were either treated laparoscopically or robotically. Based on their findings, the robotic-treated group had lower mean costs of index admissions (USD 24,983 +/− 18,329 vs. open 32,983 +/− 31,983 *p* < 0.001). Additionally, it should be noted that length of stay over 7 days and anastomosis of hepatic duct to the gastrointestinal tract are strong risk factors for readmission and mortality. Robotic-assisted liver surgery has been known to have a decreased length of stay when compared to laparoscopic and open surgery (robotic 4.5 +/− 3.8 vs. laparoscopic 6.8 +/− 6 vs. open 7.6 +/− 7.7 days *p* < 0.01), and has been shown a lower likelihood of readmission within 45 days (7.9% robotic vs. 13% lap vs. 13.8% open, *p* < 0.05). The robotic group is also associated with lower rates of mortality (0.5% vs. 4% vs. 3.1%, *p* < 0.05) [34].

Another meta-analysis by Papadopoulou et al. (2022) [35] compared the short-term outcomes between robotic liver resection and open liver resection. Their study compiled worldwide research, including five studies from Asia, four from Europe, four from America, and one from multiple countries. Their meta-analysis included 13 non-randomized retrospective and one prospective study between 2014 and 2022. Indications for resection include benign and malignant liver tumors, with benign liver conditions including hepatolithiasis. Of these indications, major hepatectomies made up the majority, with 41.2% treated with robotic-assisted surgery and 35.2% in the open group. There was no difference in the number of tumors, with a mean of 1.45 for the robot group vs. 1.39 for the open group [35]. Their study concluded that the patients in the robotic group had lower morbidity, a shorter hospital stay, and less intraoperative blood loss. It should be noted that intraoperative transfusion was more likely to occur in the open liver surgery group (18.9%) when compared to the robotic group (10.6%). Morbidity rates were also lower for the robotic group at 19% compared to the group at 32.1%.

The postoperative outcomes of robotic hepatectomy mortality are 0.4% compared to 0.69% for open hepatectomy. Morbidity rates were divided into two categories based on Clavien–Dindo scores. A score of I–II indicated minor liver disease, while a score of III–IV indicated more severe disease. In total, 13.7% of patients with a Clavien–Dindo score of I–II experienced postoperative morbidities in the robotic group, while 25% of patients with the same Clavien–Dindo score experienced morbidities in the open group. In patients with a Clavien-Dindo score of III–IV, 4.5% experienced postoperative morbidities in the robotic group vs. 6.8% in the open group. The overall morbidity rate was 19% in the robotic group and 32.1% in the open group. Of the postoperative complications, 2.2% in the robot group experienced bile leakage, while 3.3% experienced bile leakage in the open group. However, the robotic group had a significantly higher operative time, with a mean difference of −58.8 min. There were no statistically significant differences between the two groups regarding blood transfusion requirements, Ro resection, and mortality rates; however, patients in the robotic liver resection group did have better outcomes than the open group. This meta-analysis shows that the short-term outcomes of robotic-assisted liver surgery are safe and effective compared to their open liver surgery counterparts. Robotic-assisted liver surgery may be more advantageous since this approach has been shown to have fewer complications (13.7% RLR vs. 25% OLR) and, therefore, less morbidity without sacrificing oncologic outcomes. Initially, negative resection margin and postoperative mortality had better outcomes in the robotic group, but the results were not statistically significant. However, the low conversion rate (mean = 5%, ranging from 0 to 10%) and conclusive results support its efficiency and safety. A lower complication rate could be associated with a shorter length of stay, hence earlier chemotherapy induction, which could lead to favorable oncologic outcomes [36]. Papadopoulou et al. (2022) [35] concluded that the robotic approach is superior to traditional open liver resection in terms of short-term outcomes.

All of these analyses can also be seen in Table 1.

## 3. Conclusions

The robotic surgical system has infiltrated the field of hepatic surgery. Many studies compare its safety, efficacy, and cost-effectiveness to open or laparoscopic approaches, with a growing number of liver surgeons implementing this new technique in complex liver resections. Robotic-assisted liver resections are advantageous over conventional laparoscopy, with lower perioperative morbidity and shorter hospital stay, which can help offset the substantial costs of robot acquisition and maintenance [37]. Oncologic outcomes of robotic hepatectomy appear to be equivalent to laparoscopic and open hepatectomy for appropriately selected patients; therefore, careful patient selection is essential to maximize positive outcomes [30]. Also, more studies need to be performed to quantify the additional benefits of robotic surgery and evaluate long-term outcomes. Despite the addition of endowrist technology and fluorescence guidance, the current Da Vinci robotic system is still limited by the number of devices used for parenchymal transection and still lacks haptic feedback. Despite these setbacks, the use of robotics is expected to rise in hepatobiliary surgery to treat benign diseases and malignant neoplasms. Surgical education must evolve alongside the rapidly changing operative environment, with innovations like online resources and robotic technology playing a growing role. A recent study by Green et al. [34] from the University of California, San Francisco, examined how robotic surgery impacts resident training and identified four key challenges: (1) dual skill learning (surgical and technological), (2) the timing of robotic exposure, (3) the comparison of surgical modalities, and (4) communication and dual console use [38]. Trainees must learn both surgical techniques while also learning about the robotic system, which can cause cognitive overload. Teaching robotic skills outside the operating room through simulations may improve learning outcomes. There is also inconsistency across institutions when residents are introduced to robotics. More research is needed to assess standardized approaches like pre-fellowship robotic courses. Educators and surgeons disagree on whether robotic surgery aligns more with open or laparoscopic techniques. Emphasizing both similarities and differences can help trainees grasp multiple minimally invasive approaches. The robotic environment requires precise verbal instruction, especially with the dual console setup. Effective communication is essential, since physical proximity is reduced in robotic surgery. This study also acknowledges limitations such as a narrow participant pool, absence of trainee perspectives, and potential respondent bias due to the data being collected at a single national conference. Further research is needed to confirm and generalize these findings. Differences in complication rates among open, laparoscopic, and robotic surgeries are largely influenced by surgeon experience, often more so than the limitations of the robotic system itself. Studies show that complication rates are higher early in a surgeon’s robotics learning curve, especially for those trained post-residency, and tend to decrease with experience. This suggests that many complications stem from inexperience rather than flaws in the technology. Transitioning from open or laparoscopic techniques to robotics also requires skill adaptation, increasing early error risk, especially given inconsistent robotic training during residency [34]. While robotic systems offer benefits like improved dexterity and visualization, these advantages depend on proper training. Current data often overlook factors like surgeon experience and case complexity, making it hard to isolate the system’s true impact. As robotics training becomes more standardized, complication rates should better reflect the platform’s real potential.

We expect to see a rise in the use of robotic technology in liver surgery as liver surgeons’ experiences increase and more tools are implemented, and more favorable results will likely be presented in the literature [38].

## Figures and Tables

**Table 1:** Cumulative meta-analysis findings.

Year Published	Author	# Of Studies		Robotic	Laparoscopic	Open
2019	Machairas et al. [30]	10	Morbidity rate	15.5%		22.2%
			Major adverse events	3.2%		5.5%
			Minor adverse events	11%		15.9%
			Postoperative complications (i.e., bile leaks)	2.2%		2.7%
			Bleeding	2.2%		2.9%
2019	Fruscione et al. [32]		Readmission to ICU	43.9%	61.2%	
2019	Cortolillo et al. [33]		Length of hospital stay	4.5 ± 3.8	6.8 ± 6	7.6 ± 7.7
			Mortality	0.5%	4%	3.1%
			Readmission within 45 days	7.9%	13%	13.8%
2021	Ziogas et al. [31]	7	Overall Complication Rate	18.4%	28.3%	
			Overall Mortality Rate	0%	0.3%	
			Transfusion Rate	18.4%	14%	
			Conversion to open	3.7%	6.4%	
			Margin positive resection	6.1%	10.1%	
2022	Papadopoulou et al. [35]	14	Intraoperative transfusion rate	10.6%		18.9%
			Overall morbidity rate	19%		32.1%
			Overall mortality rate	0.4%		0.69%
			Postoperative complications (i.e., bile leaks)	2.2%		3.3%
			Major complications	4.5%		6.8%
			Minor complications	13.7%		25%

## Data Availability

The original contributions presented in this study are included in the article. Further inquiries can be directed to the corresponding author.
